# Cases of Lock Jaw and Tetanus

**Published:** 1809-08

**Authors:** John Howship

**Affiliations:** Surgeon


### CASES OF LOCK JAW AND TETANUS. By JOHN HOWSHIP, Surgeon.

### *Case of Tetanus, in which Amputation failed in checking the fatal Progress of the Disease.*

UPON the memorable 21st of October, 1805, I. Kelly, of H. M. S. Mars, was struck by a heavy shot, which in passing through the sides of the ship produced a compound fracture of the leg. From the apparent state of the wound, hopes hopes were entertained that the limb might be saved, particularly as the loss of blood had not been considerable. No remarkable event occurred earlier than the 10th day, at which period he complained of stiffness and difficulty in moving his jaws, as well as of the pain in deglutition that generally takes place in the early stage of this formidable disorder.

A decided and vigorous plan of treatment was not immediately adopted, and nothing of consequence was done till the evening of the 13th day, when the rubbing in of the mercurial ointment was commenced. The frictions were ordered to be repeated every hour, and the ointment was rubbed in over the spine and limbs.

Upon the 14th day the pulse was very small and much hurried. The twitches of spasm were frequent, though apparently not very severe. The jaws were so firmly locked as to render the introduction of any solid food quite impracticable.

It was very evident that the exciting cause of this unfortunate man's distress was his wound; it was therefore determined to afford every possible chance for the preserving of life by the removal of the diseased member. The operation was performed at two in the afternoon, the leg being taken off above the knee.

The probability of the ultimate success of the operation was not at first doubtful, for even before the stump was dressed the man spake better than before, said he was greatly relieved, and expressed the clearest conviction that he should now recover. The most remarkable circumstance, however, was his finding that the power of separating his jaws to a certain degree, had returned to him again; he tried to open his mouth, and was observed to separate his teeth so well as to allow a table spoon to pass freely, though before the removal of the limb his jaws were constantly and closely locked.

Every one present concurred in the opinion that he was wonderfully relieved by the operation that had been performed, and the man himself, soon after he was composed and quiet in bed, told some of his companions that he was in every respect much better.

From the time of the amputation, the spasms affecting the voluntary muscles of the system left him; these constitute perhaps the chief peculiarity, and certainly are productive of the highest degree of torment to those who are afflicted with this malady; these were very frequent till the limb was removed, but from the moment when the division of the soft parts was completed, these spasms ceased altogether and never afterwards returned.

He continued to be, as the attendants state, much better, more cheerful and lively than he was before, till about five o'clock in the afternoon, when he was seized with a sort of convulsive tremor, a general state of vibration of the whole system, resembling the cold fit of an ague. This had continued for some short time, when an impediment to his breathing seemed to arise about the throat; he was now in a state of approaching suffocation, and very soon afterwards sunk into the arms of death.

On the day this patient was first attacked, the 10th after the action, he was seized in a very singular manner; to all appearance he fell into a swoon, in which he was so perfectly without motion for an hour and a half, that it was doubted whether he would recover from it; the heart could not be felt to beat, the breast remained unmoved, nor could the halitus of respiration be perceived, upon placing a looking glass over the mouth.

Upon coming to himself he recovered his faculties quickly, and told his attendants that he knew he should very soon die, for that he had seen his father and his friends, while in the apparent state of swooning. His lucid interval was of very short duration, and was succeeded by a state of delirium, in which he fell into frequent fits of laughter, abusing those that were near him, and spitting in the faces of such as were rendering him assistance.

The senior medical officer of the hospital declared that he was convinced this man died from debility, and that only.

*A Case of Tetanus, in which the Close of the Scene resembled that of the Case last described, although the Circumstances of it were essentially different.*

W. Connars, aged 29, a seaman belonging to H. M. S. Mars, was severely wounded in the back by a splinter from the ship's side. It was not before the 11th day that symptoms of Lock Jaw first appeared. On the 12th, mercury was introduced both by internal and external exhibition. Upon the 13th day the system remained unaffected by the mode of treatment. At this time the spasms about the jaws were frequent and distressing. The pule was little quickened, but was firm and throbbing.

From the muscles about the face, neck, and chest, the spasmodic affection extended itself to all parts of the body and limbs.

Upon

Upon the 14th day he was in every respect worse; his mouth was by this time somewhat affected, but the disease had become much more severe, being established in its most aggravated form. The sufferings of this poor man continued, with short intervals of alleviation, till the 16th day, when towards the evening he fell into the same state of violent trembling that occurred in the case last mentioned; the excessive violence of the shaking fit gradually subsided, all motion of the body ceased, and in a tranquil state of insensibility he soon afterwards breathed his last.

### *A Case of Lock Jaw, in which the Course of the Disease was arrested and the Complaint cured, by Amputation.*

On board one of the hospital ships, lying in the Rio de la Plata, after the disgraceful and unfortunate attack upon Buenos Ayres, in July, 1807, was William Taylor, aged 25, under the care of Mr. Burnal, Apothecary to the forces. He was a thin young man, and had been wounded on the 4th of July, by a ball which shattered the elbow joint. The mischief however was not so extensive as to preclude all hope that the limb might be saved, no operation therefore was proposed, but every proper means adopted for the preventing an excessive degree of inflammation.

It was in this case as late as the 22d day before the patient first perceived a stiffness about the jaws, which prevented him from opening his mouth so well as usual.

Some degree of pain followed after the stiffness, but the progress of the complaint was so very slow that it was three days after he had first perceived the stiffness, before he made mention of his sensations to the surgeon on board, who upon examination found that his jaws were stiff, but by no means closely locked.

The wound upon the 25th day was productive of great disturbance to the system, being exceedingly painful, very tumid and highly inflamed, besides which there had been large and repeated collections of matter.

At this time amputation was proposed, being considered to afford a fairer prospect of recovery, than any course of medical treatment could ensure. The operation was unattended by any peculiar circumstances. From the time of the limb being removed, he began to mend, and continued to improve daily. He found himself gradually recover, and by the 29th day, being the fourth after the operation, he was able to open his mouth very well, and felt no more pain, and very little remaining stiffness.

 About

About a week after the operation he complained of a very severe pain in the chest, which, however, yielded to the common treatment, and the man perfectly recovered.

*Scarbro', June* 19, 1809.

( To be continued. )

